# Temporal Dynamics of Ensemble Face Representations Under Magnocellular- and Parvocellular-Biased Visual Inputs: A Time-Resolved EEG Decoding Study

**DOI:** 10.3390/brainsci16090996

**Published:** 2026-09-20

**Authors:** Tianbo Li, Huanxiang Sun, Yi Jiang, Xiuling Zhang

**Affiliations:** 1 School of Psychology, Northeast Normal University, Changchun 130024, China; litbpsy@nenu.edu.cn (T.L.); hxsunpsy@nenu.edu.cn (H.S.); 2 Jilin Provincial Key Laboratory of Cognitive Neuroscience and Mental Health, Changchun 130024, China; 3 State Key Laboratory of Cognitive Science and Mental Health, Chinese Academy of Sciences, Beijing 100101, China; yijiang@psych.ac.cn; 4 Department of Psychology, University of Chinese Academy of Sciences, Beijing 100049, China

**Keywords:** ensemble perception, average representation, magnocellular pathway, parvocellular pathway, EEG, multivariate pattern analysis

## Abstract

**Highlights:**

**What are the main findings?**

- Gender information from M-biased face ensembles was reliably decodable early in processing and showed significant early temporal generalization.
- Under M-biased stimulation, ensemble gender information generalized to single-face judgments during late processing.

**What are the implications of the main findings?**

- Ensemble face-gender processing shows a multistage temporal organization, with gender information becoming decodable early and remaining available at later stages.
- Shared task-related information between ensemble and single-face judgments during late processing suggests a potential link between ensemble representations and item-level gender information.

**Abstract:**

Background/Objectives: Ensemble perception enables the visual system to rapidly extract summary statistics from multiple objects. However, the temporal dynamics of ensemble average representations and their dependence on magnocellular-biased (M-biased) and parvocellular-biased (P-biased) visual information remain unclear. This study examined when ensemble face-gender information could be decoded under these two stimulus conditions. Methods: Twenty-four healthy adults performed a two-alternative forced-choice gender judgment task with neutral face ensembles and single faces. M-biased stimuli were low-luminance-contrast grayscale faces, whereas P-biased stimuli were isoluminant red–green faces. Time-resolved multivariate pattern analysis, temporal generalization, and cross-condition generalization characterized gender-related EEG dynamics. Results: Behaviorally, responses were faster for P-biased than M-biased stimuli and for single faces than ensembles, with no significant accuracy differences. Balanced Integration Scores were significantly higher for P-biased than M-biased stimuli in both ensemble and single-face conditions. M-biased ensemble gender information was reliably decodable during early (95–300 ms) and late processing (775–930 ms), with significant early temporal generalization. P-biased face ensembles showed no significant cluster-corrected time-resolved decoding but exhibited significant temporal generalization during middle-to-late processing. Under M-biased stimulation, ensemble gender information generalized to single-face judgments from 690–995 ms. Conclusions: Ensemble face-gender information showed a multistage temporal organization. M-biased ensemble information became decodable early. Late cross-condition generalization further indicated that ensemble and single-face judgments shared gender-discriminative task information. These findings characterize the temporal evolution of ensemble gender information under pathway-biased visual stimulation and provide a temporal perspective on ensemble perception.

## 1. Introduction

As we pass a mass of flowers in bloom, we do not need to examine the color, size, and degree of blooming of each flower individually. Instead, a brief glance is often sufficient to grasp the overall color, density, and general state of bloom. Such efficiency is particularly remarkable because everyday visual scenes contain far more information than can be processed in detail, given the limited capacity of attention and visual working memory. If perception depended on the precise encoding of each individual object, it would be difficult to explain how the visual system can so rapidly extract the overall properties of a scene from rich and redundant visual input. Ensemble perception addresses this challenge by summarizing multiple similar items through statistical properties such as their mean and variability. It thereby supports efficient scene representation at minimal cognitive cost [1]. Among these statistical properties, average attributes have received the greatest attention [2]. However, the neural mechanisms and temporal dynamics underlying average representations remain poorly understood.

First, although behavioral studies demonstrate that the visual system rapidly and accurately extracts average features from ensembles [3,4,5,6], the underlying neural mechanisms remain insufficiently understood, and the temporal dynamics governing their emergence and evolution require further investigation. Electrophysiological findings suggest that average representations can form rapidly during early visual processing. For instance, event-related potential (ERP) studies using oddball paradigms observed effects related to ensemble average information around 100 ms [7,8], and a magnetoencephalography (MEG) study reported neural discrimination between ensemble and single stimuli as early as 68 ms [9]. Conversely, recent studies employing neural decoding techniques suggest that average representations directly associated with behavioral performance emerge at later stages. An MEG study utilizing inverted encoding models (IEMs) demonstrated that neural activity representing ensemble averages primarily occurred between 370 and 700 ms, with decoding peaks correlating significantly with behavioral accuracy [10]. Similarly, an electroencephalography (EEG) study found that IEM signals representing mean orientation did not correlate with explicit behavioral responses until a late processing stage around 600 to 700 ms [11]. Although previous studies observed ensemble-related neural activity across early and late time windows, discrepancies in experimental paradigms and analytical metrics prevent a coherent interpretation of the full temporal trajectory of average representations. These early and late neural signatures may reflect distinct stages in the dynamic construction of ensemble representations, a possibility that warrants systematic examination within a unified experimental design.

Second, understanding the temporal dynamics of early and late ensemble processing requires further clarification of the visual inputs that may support the formation of ensemble perception. Previous studies suggest that coarse global information can be rapidly extracted and conveyed to higher-level brain regions before detailed analysis is complete, thereby facilitating subsequent perception [12,13]. Further evidence links this rapid processing to magnocellular input, as M-biased stimuli preferentially engage orbitofrontal regions involved in early top-down facilitation [14]. Functionally, the M pathway has high temporal resolution and is particularly sensitive to low luminance contrast, low spatial frequencies, and rapid transient changes, making it well suited for the rapid extraction of coarse global structure from multiple simultaneously presented stimuli. In contrast, the P pathway provides higher spatial and chromatic resolution and better supports the subsequent analysis and integration of visual details [15]. These properties make M-pathway involvement a plausible account of early ensemble perception. However, the respective contributions of M- and P-biased visual inputs to ensemble perception remain debated. Functional magnetic resonance imaging (fMRI) findings showed that ensemble emotion judgments elicited stronger activation in dorsal stream regions, such as the intraparietal sulcus and superior frontal gyrus, than individual emotion judgments [16]. Subsequent MEG evidence revealed dorsal stream activity as early as 68 ms post-stimulus onset, indirectly suggesting that the M pathway mediates the early processing of ensemble faces [9]. Conversely, suppressing M-pathway activity through a flicker adaptation paradigm did not impair ensemble judgment precision, indicating that fine-grained visual information may also contribute to ensemble perception [17]. Because these previous studies relied on indirect inferences from dorsal activations or psychophysical adaptation rather than directly comparing physically controlled M-biased and P-biased visual inputs, systematic investigations under rigorous stimulus control are needed. Such work must determine whether M- and P-biased inputs play dissociable roles across different temporal stages and, in particular, whether M-biased information selectively supports the initial formation of average representations.

Methodologically, biasing visual processing toward M- and P-related inputs provides a useful approach for testing these temporal hypotheses. Previous studies often mapped low spatial frequencies (LSF) and high spatial frequencies (HSF) onto the magnocellular and parvocellular pathways, respectively. For instance, Zhao et al. [18] demonstrated that emotion recognition from crowd faces was faster and more accurate for LSF stimuli, whereas HSF crowd faces selectively engaged ventral visual areas such as the fusiform gyrus. However, because the spatial frequency tuning curves of M and P neurons overlap substantially, LSF information cannot be treated as an exclusive proxy for M-pathway input [19], nor do discrete spatial frequency bands cleanly separate the two streams [20]. Relying solely on spatial frequency manipulations is therefore insufficient to isolate their distinct contributions. Psychophysical manipulations of luminance and chromatic contrast can bias visual processing: low-luminance-contrast grayscale stimuli are used to favor M-related processing, whereas isoluminant red–green stimuli are used to favor P-related processing. Heterochromatic flicker photometry (HFP) estimates isoluminance by rapidly alternating two chromatic stimuli and adjusting their relative luminance until perceived flicker is minimized [21]. This procedure has been applied to red–green object stimuli [14], face stimuli [22], and hierarchical stimuli [23] to obtain participant-specific isoluminance settings for pathway-biased visual stimulation. Related manipulations have been used in studies of object recognition [14], global versus local processing [24], and facial threat cue perception [25]. These stimuli provide a basis for comparing the temporal availability of ensemble-related information under different visual input conditions, while supporting inferences about relative pathway biases.

Furthermore, using facial stimuli to investigate pathway biases requires controlling for potential confounds associated with emotional processing. Although crowd faces displaying fearful expressions exhibit an LSF recognition advantage [18], individual emotional faces, particularly threat-related expressions, are also sensitive to LSF and M-biased visual information. Prior studies have established that LSF fearful faces trigger robust amygdala activation [26], enhance neural responses in the fusiform cortex [27], and provide critical diagnostic cues for threat classification [25,28,29]. Consequently, early or low-frequency processing advantages observed in emotional ensemble tasks may reflect a confluence of ensemble statistical extraction and threat-specific emotional processing. Employing neutral face gender morphs devoid of affective valence mitigates interference from emotion-driven pathways, allowing for a cleaner assessment of how ensemble average representations depend on pathway-biased visual inputs.

To address these empirical and theoretical questions, the present study manipulated luminance and chromatic contrast to compare the temporal dynamics of ensemble processing under M- and P-biased visual inputs, combining high-temporal-resolution EEG recordings, time-resolved multivariate pattern analysis (MVPA), and temporal generalization. Participants performed a two-alternative forced-choice gender-judgment task on neutral face ensembles and single faces presented under M-biased (low-contrast grayscale) and P-biased (isoluminant red–green) conditions. This design was intended to characterize when ensemble gender information becomes neurally available under M-biased and P-biased visual inputs and how it is organized over time under these conditions. We expected ensemble gender information to become decodable during early visual processing under M-biased stimulation. P-biased ensemble information was also expected to show temporal generalization during middle-to-late processing. These predictions were used to test whether ensemble face-gender information exhibits a multistage temporal organization under pathway-biased visual stimulation.

## 2. Materials and Methods

### 2.1. Participants

An a priori sample-size estimation was conducted in G*Power 3.1 to ensure adequate statistical power. With a medium effect size (effect size *f* = 0.25), a significance level of *α* = 0.05, and statistical power of 1 − *β* = 0.80, the minimum sample size required for a repeated-measures ANOVA was 24 participants [30]. Twenty-seven healthy undergraduate and graduate students were recruited. Three participants were excluded because extensive EEG artifacts left fewer than 200 artifact-free trials in at least one experimental condition after preprocessing (see Section 2.4). The final sample comprised 24 participants (8 men, 16 women; age *M* = 21.67 years, *SD* = 2.30). All participants were right-handed, had normal or corrected-to-normal visual acuity and normal color vision, had no history of psychiatric illness or head injury, volunteered to participate, and met the safety criteria for EEG testing. The experimental protocol was approved by the Academic Ethics Committee of the School of Psychology, Northeast Normal University, on 1 March 2023. As the committee did not assign approval numbers at the time, no approval number was initially issued; an Ethics Approval Code (202301072) was subsequently issued for this study on 27 August 2026. All participants provided written informed consent and received monetary compensation.

### 2.2. Stimuli

Faces from three female–male identity pairs (AF13 with AM29, AF21 with AM02, and AF23 with AM09) were selected from the Karolinska Directed Emotional Faces (KDEF) database [31]. In this study, facial gender refers to the perceived masculinity–femininity dimension of the morphed face stimuli. FantaMorph 5 was used to linearly morph the faces within each pair, yielding three 51-face continua from male (−25) to female (+25) in steps of 1 unit.

**Figure 1:**
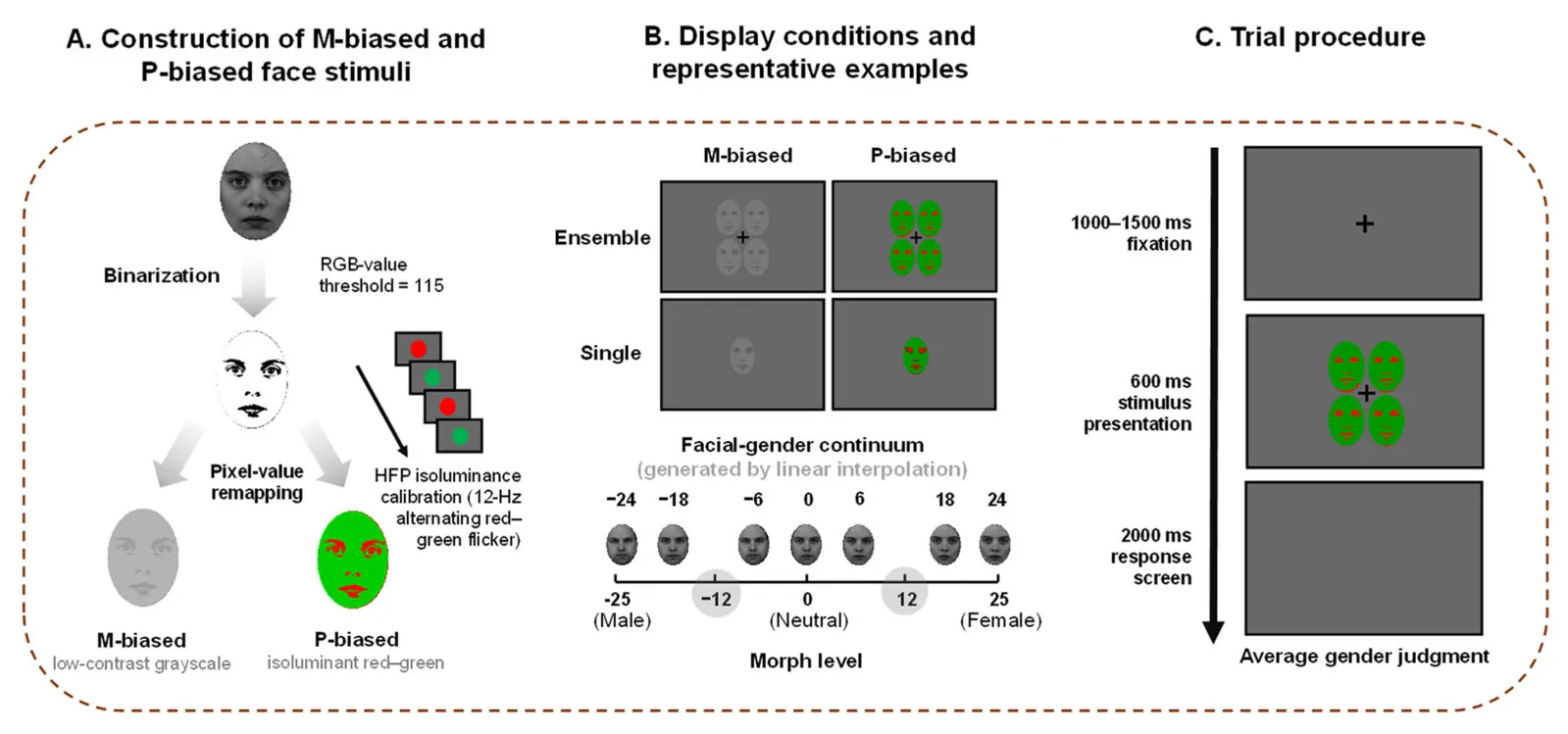
Construction of pathway-biased face stimuli, experimental conditions, and trial procedure. (**A**) Original grayscale face images were binarized using a unitless digital RGB-value threshold of 115 on a 0–255 scale. Pixel values in the resulting binary images were then remapped to low-luminance-contrast grayscale values to generate magnocellular-biased (M-biased) stimuli or to group-calibrated isoluminant red–green values to generate parvocellular-biased (P-biased) stimuli. Red–green isoluminance was estimated using heterochromatic flicker photometry (HFP) at 12 Hz; the calibrated red and green values were RGB = [200, 0, 0] and RGB = [0, 78, 0], respectively. (**B**) Representative ensemble and single-face stimuli under the M-biased and P-biased conditions. The facial gender continuum was generated by linear interpolation and ranged from −25 (male endpoint) to +25 (female endpoint), with 0 representing the neutral midpoint. Single-face stimuli were selected at morph levels −12 and +12. The illustrated masculine and feminine ensembles comprised morph levels −24, −18, −6, and 0 (mean morph level = −12) and 0, 6, 18, and 24 (mean morph level = +12), respectively. (**C**) Each trial began with a fixation cross presented for 1000–1500 ms, followed by an ensemble or single-face stimulus presented for 600 ms and a response screen presented for 2000 ms. Participants judged whether the average gender of the ensemble, or the gender of the single face, was more masculine or more feminine than the neutral midpoint.

To standardize face luminance and contrast, all faces were adjusted using MATLAB, and binary face images were generated by thresholding at an RGB value of 115. Before the EEG experiment, eight participants who subsequently took part in the formal EEG study completed an HFP task to obtain a display-specific red–green isoluminance setting. The calibration program was run on the laboratory display. Red and green stimuli alternated rapidly at 12 Hz, with the red value fixed at RGB = [200, 0, 0]. Participants adjusted the green-channel intensity using a continuous slider until flicker caused by the red–green luminance difference was minimized and the two colors appeared perceptually fused. The observer-level green-channel settings showed limited between-observer dispersion (*M* = 78.00, *SD* = 2.45, range = 74–82), with all eight settings falling within four RGB units of the group mean. Given the limited between-observer dispersion, the group mean was adopted as the display-specific calibration value for generating a common P-biased stimulus set. Applying one common calibration value maintained an identical physical red–green mapping across all EEG participants and P-biased conditions. The final values used to generate the P-biased stimuli were RGB = [200, 0, 0] for red and RGB = [0, 78, 0] for green (see Figure 1A).

Stimuli were presented on a neutral gray background. Specifically, the arithmetic mean of the isoluminant red and green channel values was first calculated, and this value was assigned to the red, green, and blue channels to obtain a gray background of RGB = [139, 139, 139]. The low-contrast threshold was set to 5% Weber contrast, and the RGB value of the light-gray face foreground was calculated from the gray background RGB value. On the basis of the resulting red, green, dark-gray, and light-gray values, three sets of linearly morphed face sequences were generated for the M-biased and P-biased conditions. Each face member subtended a visual angle of 3.04° × 2.33°. According to a behavioral pilot experiment, the male (−12) and female (+12) faces in the linear face sequence were defined as the gender values for ensemble and single-face stimuli. To ensure sufficient variation among members within each ensemble stimulus, the difference between any two members was no less than 6 units, allowing participants to distinguish the ensemble members. To control for position effects, all permutations of the four ensemble members across four spatial locations were enumerated, yielding 4! = 24 arrangements. Across all arrangements, each member appeared six times at each spatial location, balancing the correspondence between member identity and spatial position.

### 2.3. Experimental Procedure

The experiment used a 2 (stimulus bias: M-biased/P-biased) × 2 (stimulus category: ensemble/single) within-participants design. Participants completed a two-alternative forced-choice task (2AFC) in which they judged the gender of an ensemble or a single face on each trial. Dependent variables included behavioral accuracy, reaction time, and EEG data recorded during gender judgment. The experiment included four conditions: M-biased ensemble, M-biased single, P-biased ensemble, and P-biased single. Before the formal experiment, participants completed 30 practice trials to ensure that they understood the task procedure. The formal experiment comprised 1152 trials, with 288 trials in each condition and 576 trials each for ensemble and single stimuli. To reduce fatigue during the extended session, participants took a short break after every 40 trials. The presentation order of ensemble and single stimuli was balanced using an ABBA sequence.

The experiment was programmed in E-Prime 2.0 and conducted in a sound-attenuated, enclosed laboratory with constant illumination. The computer was a Dell OptiPlex 755 (Dell Inc., Round Rock, TX, USA). The display was 27 inches, with a refresh rate of 60 Hz and a resolution of 1920 × 1080 pixels. During the experiment, participants placed their chin on a chin rest so that their eyes were 60 cm from the display, and they responded using a standard keyboard. The experiment was conducted in a single-participant electromagnetically shielded room to reduce environmental noise and electrical interference. Each trial began with a fixation point presented for 1000–1500 ms, followed by an ensemble or single face presented for 600 ms and then a blank screen presented for 2000 ms. Participants responded during the blank screen. They pressed key “1” if they judged the ensemble or single face to be more masculine than a neutral face, and key “2” if they judged it to be more feminine. The next trial began after the blank screen ended (see Figure 1C). The 600-ms duration was identical across all four conditions and was chosen to maintain visual input during early ensemble extraction and much of the subsequent middle-to-late processing period [11]. It also avoided substantially longer exposure durations, as previous work found no further significant improvement in facial ensemble discrimination at longer durations [6].

### 2.4. EEG Recording and Preprocessing

EEG data were recorded using a Neuroscan 64-channel Ag/AgCl electrode EEG amplifier (SynAmps2, Neuroscan, Charlotte, NC, USA), with electrodes positioned according to the extended international 10–20 system. Vertical electrooculography (VEO) was recorded using electrodes placed 1.5 cm above and below the left eye, and horizontal electrooculography (HEO) was recorded using electrodes placed 1.5 cm lateral to the outer canthi of both eyes. Data were sampled at 1000 Hz and online band-pass filtered from 0.01 to 100 Hz. The left mastoid (M1) served as the online reference electrode, and the forehead electrode (GND) served as ground. Electrode impedances were kept below 5 kΩ throughout recording.

All EEG data were preprocessed in MATLAB R2024a (MathWorks, Natick, MA, USA) using the EEGLAB v2024.2 toolbox [32]. First, five external electrodes (M1, CB1, CB2, HEO, and VEO) were removed from the raw data, leaving 60 scalp electrodes and the right mastoid M2 channel. Continuous EEG data were then filtered sequentially with zero-phase finite impulse response (FIR) filters using a Hamming-window design: a 0.1-Hz high-pass filter, a 70-Hz low-pass filter, and a 49–51-Hz notch filter to remove 50-Hz line noise. All filters were applied bidirectionally to achieve zero-phase distortion [33]. Next, continuous EEG data were epoched from −1000 to 2000 ms relative to stimulus onset, and baseline correction was applied using the 100 ms before stimulus onset (−100 to 0 ms). After epoching and baseline correction, bad epochs and bad channels were manually screened, and data segments or channels with obvious drift, noise, or other artifacts were removed or interpolated. The data were then rereferenced to the average of the bilateral mastoids, using M2 together with the M1 online-reference recording. Independent component analysis (ICA) was performed using the extended Infomax algorithm (runica [34]). Independent components were automatically classified with ICLabel v1.7 [35], and ocular components (Eye probability ≥ 0.9) and muscular components (Muscle probability ≥ 0.9) were removed. Participants were excluded if fewer than 200 artifact-free trials remained in any of the four experimental conditions after preprocessing. This criterion was applied before selecting correct-response trials for decoding, with counts pooled across male and female trials within each condition. Among the 24 retained participants, 4526 of 27,648 epochs (16.37%) were rejected because of excessive drift, noise, or other artifacts, leaving 23,122 artifact-free epochs (83.63%) after preprocessing.

### 2.5. Data Analysis

#### 2.5.1. Behavioral Analysis

Behavioral accuracy and mean reaction time (RT) for correct-response trials were analyzed separately using 2 (stimulus bias: M-biased vs. P-biased) × 2 (stimulus category: ensemble vs. single-face) repeated-measures ANOVAs. To supplement these analyses with a measure jointly incorporating response speed and accuracy, we calculated the Balanced Integration Score (BIS) following Liesefeld and Janczyk [36]. For each participant and condition, accuracy and mean correct-response RT were *z*-standardized across all 96 participant-by-condition observations, and BIS was calculated as BIS = *z*(accuracy) − *z*(RT), with higher values indicating a more favorable combination of accuracy and response speed. BIS was analyzed using the same 2 × 2 repeated-measures ANOVA. Supplementary two-tailed paired-samples *t* tests compared M-biased and P-biased BIS values separately for ensemble and single-face displays, with Bonferroni correction across the two comparisons. Partial eta squared (*η_p_*^2^) and Cohen’s *d_z_* were reported as effect-size measures, with *α* = 0.05.

#### 2.5.2. Time-Resolved Decoding

To examine whether face gender information could be decoded from EEG signals under different stimulus-bias and stimulus-category conditions, time-resolved binary classification of male versus female faces was performed separately for the M-biased ensemble, M-biased single, P-biased ensemble, and P-biased single conditions. MVPA was performed using EEG signals from a prespecified set of 60 scalp electrodes. EEG decoding analyses were implemented using MNE-Python version 1.12.1 [37] and scikit-learn version 1.8.0. Epoched EEG data from each participant were resampled to 200 Hz, time-locked from 100 ms before to 995 ms after stimulus onset, and baseline-corrected using the −100-to-0-ms prestimulus interval. Because the information to be decoded was subtle, only artifact-free trials with correct behavioral responses that had been retained in the preprocessed, condition-specific EEG files were included in the decoding analysis, and pseudotrial averaging was used to improve the signal-to-noise ratio. Within each participant, experimental condition, and gender category, trials were randomly partitioned without replacement into nonoverlapping groups of four and averaged to form pseudotrials. Thus, within each randomization, each raw trial contributed to at most one pseudotrial. Pseudotrial counts were equated between the two gender categories by retaining, for both categories, the number of pseudotrials available in the category with fewer pseudotrials and discarding surplus pseudotrials from the larger category after randomization. Pseudotrial randomization and decoding were repeated 50 times.

Decoding was performed using a logistic regression classifier (LogisticRegression). The regularization parameter was set to *C* = 0.001, and balanced class weights were applied. Within-condition decoding used stratified cross-validation with a target of five folds. When fewer than five pseudotrials were available in either gender category, the number of folds was reduced to the number of pseudotrials available in the smaller category. Nonoverlapping pseudotrials were assigned to the training and test folds. Within each cross-validation fold, feature-standardization parameters were estimated exclusively from the training set and then applied to the held-out test set. At each classifier-center time point, EEG activity from the 60 electrodes within a centered 50-ms temporal window was concatenated to form the feature vector. At a sampling rate of 200 Hz, each window contained 11 samples and extended approximately 25 ms on either side of its nominal center. The samples within each window were retained as separate features rather than temporally averaged. A separate classifier was fitted at each center time point, yielding time-varying decoding performance. Classification performance was quantified as the area under the receiver operating characteristic curve (AUC), with a chance level of 0.5. AUC values were averaged across cross-validation folds within each randomization and subsequently averaged across the 50 pseudotrial randomizations to obtain each participant’s time-resolved decoding curve. Group-level statistical analyses were conducted on the native 200-Hz time grid from −100 to 995 ms, without temporal interpolation or statistical smoothing. Participant-level AUC values were centered by subtracting 0.5, and a one-sample *t* statistic was calculated across participants at each time point. A one-tailed, one-sample cluster-based permutation test was used to determine whether decoding performance exceeded chance. A total of 5000 permutations were performed, and the cluster-forming threshold corresponded to *p* = 0.05. Cluster-level *p* values controlled the family-wise error rate across time within each decoding condition; no additional correction was applied across the four decoding conditions.

Exploratory neural–behavioral analyses assessed the association between decoding AUC and behavioral accuracy across the 24 participants. Pearson correlations were calculated at each time point separately for the four experimental conditions. For visualization, time points showing nominally significant pointwise correlations were selected for scatterplots, using a two-sided threshold of *p* < 0.05 without correction for multiple comparisons across time and conditions.

#### 2.5.3. Cross-Condition Generalization Decoding

To examine whether gender-discriminating information generalized across stimulus configurations, ensemble-to-single cross-condition decoding was performed separately under M-biased and P-biased stimulation. The analysis used the same EEG epochs, 50-ms sliding windows, 5-ms step size, and full set of 60 scalp electrodes as the within-condition decoding analysis.

Within each window, voltage values from all scalp electrodes and temporal samples were flattened and concatenated into a 660-dimensional spatiotemporal feature vector. An L2-regularized logistic regression classifier was trained to discriminate masculine from feminine ensemble pseudotrials and was subsequently evaluated on independent single-face pseudotrials carrying the same category labels. Thus, model training and testing were conducted across displays that differed in face number and spatial configuration while preserving the gender-classification target.

Feature-standardization parameters and classifier weights were estimated exclusively from the ensemble training data and were then applied without refitting to the single-face test data. Pseudotrial construction and cross-condition decoding were repeated 10 times, following the same randomization procedure used in the within-condition analysis. Generalization performance was quantified using AUC and assigned to the temporal midpoint of each window.

Participant-level generalization time courses were compared with chance using a one-tailed cluster-based permutation test across temporally adjacent window centers, with 5000 permutations and a cluster-forming threshold corresponding to *p* = 0.05. Cluster-level *p* values were additionally Bonferroni-corrected across the M-biased and P-biased generalization analyses.

#### 2.5.4. Temporal Generalization Decoding

Temporal generalization decoding was further used to assess whether discriminative patterns learned by the classifier at one training time could generalize to other testing times, yielding a two-dimensional training-time × testing-time decoding matrix. The analysis used epoched EEG data downsampled to 200 Hz. To improve the signal-to-noise ratio, two trials within each experimental condition and gender category were randomly averaged to create pseudotrials, after which temporal generalization decoding of gender was performed separately in the M-biased ensemble, M-biased single, P-biased ensemble, and P-biased single conditions. A 30-ms time window with a 15-ms step size was used. EEG data from 60 channels within each time window were extracted, and the data from 60 channels and 6 samples were flattened and concatenated into a 360-dimensional feature vector. A logistic regression classifier was trained within a stratified 5-fold cross-validation framework, and decoding performance was quantified using AUC. This procedure yielded a temporal generalization matrix for each participant in each condition, and group-level matrix inference was performed using a one-sample permutation test. Because AUC values are bounded, individual matrices were first clipped and logit transformed so that chance level (AUC = 0.5) corresponded to 0 in the transformed space. Multiple comparisons were then corrected using a threshold-free cluster enhancement (TFCE)-based permutation test under a two-dimensional adjacency structure (5000 permutations; one-tailed test of whether decoding performance exceeded chance), controlling the family-wise error rate across the entire temporal generalization matrix.

To provide a descriptive summary of the temporal organization of the temporal-generalization matrices, we calculated a dynamic index and a stable index. For each off-diagonal element, TG(*t_i_*, *t_j_*), participant-level differences were evaluated relative to the corresponding diagonal values, TG(*t_i_*, *t_i_*) and TG(*t_j_*, *t_j_*), using one-tailed one-sample *t* tests. An element was classified as dynamic when it was significantly lower than both diagonal values. An element was classified as stable when it was significantly above chance level (AUC = 0.5) and was not significantly lower than either diagonal value. Cells with |*t_i_* − *t_j_*| ≤ 90 ms were excluded to reduce the influence of temporal smoothing and overlapping adjacent windows. For each testing time, the proportion of eligible off-diagonal elements meeting each criterion was calculated within a centered 40 ms window. Participants were resampled with replacement 200 times, and the bootstrap standard error was displayed as the shaded area.

## 3. Results

### 3.1. Behavioral Results

**Figure 2:**
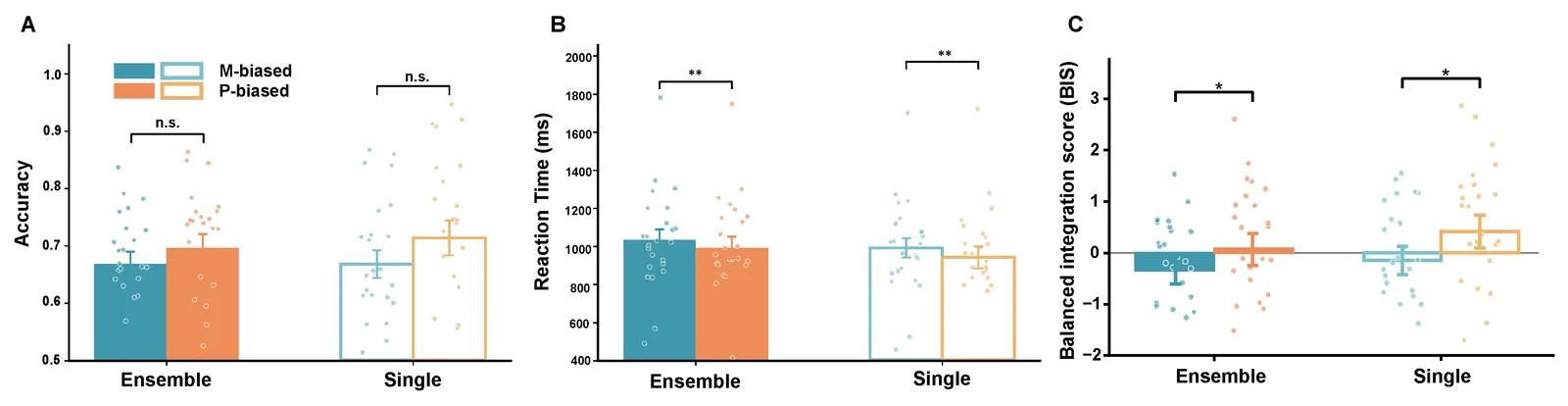
Behavioral accuracy, reaction time, and balanced integration score across conditions. (**A**) Mean accuracy. (**B**) Mean reaction time for correct responses. (**C**) Mean balanced integration score. Dots represent individual participants, and error bars indicate the standard error of the mean (SEM). * *p* < 0.05, ** *p* < 0.01; n.s., not significant.

A 2 (stimulus bias: M-biased/P-biased) × 2 (stimulus category: ensemble/single) repeated-measures ANOVA was used to test mean accuracy. The results are shown in Figure 2A. The main effect of stimulus category was not significant, *F*(1, 23) = 0.76, *p* = 0.391, *η_p_*^2^ = 0.032, indicating no significant difference in accuracy between ensemble and single stimuli. The main effect of stimulus bias was not significant, *F*(1, 23) = 3.92, *p* = 0.060, *η_p_*^2^ = 0.145, indicating no significant difference in accuracy between M-biased and P-biased stimuli (M-biased ensemble: *M* = 0.67, *SD* = 0.10; P-biased ensemble: *M* = 0.70, *SD* = 0.12; M-biased single: *M* = 0.67, *SD* = 0.12; P-biased single: *M* = 0.71, *SD* = 0.15). The interaction between stimulus bias and stimulus category was also not significant, *F*(1, 23) = 1.05, *p* = 0.316, *η_p_*^2^ = 0.043. Thus, accuracy showed no significant main effect of stimulus category or stimulus bias, nor a significant interaction between the two factors.

The same 2 (stimulus bias: M-biased/P-biased) × 2 (stimulus category: ensemble/single) repeated-measures ANOVA was used to test mean reaction time on correct-response trials. The results are shown in Figure 2B. The main effect of stimulus category was significant, *F*(1, 23) = 31.39, *p* < 0.001, *η_p_*^2^ = 0.577, with slower responses to ensemble stimuli than to single stimuli. The main effect of stimulus bias was also significant, *F*(1, 23) = 19.62, *p* < 0.001, *η_p_*^2^ = 0.460, with faster responses to P-biased stimuli than to M-biased stimuli (M-biased ensemble: *M* = 1037.11 ms, *SD* = 258.45 ms; P-biased ensemble: *M* = 996.16 ms, *SD* = 276.49 ms; M-biased single: *M* = 992.62 ms, *SD* = 247.89 ms; P-biased single: *M* = 942.98 ms, *SD* = 280.11 ms). The interaction between stimulus bias and stimulus category was not significant, *F*(1, 23) = 0.90, *p* = 0.352, *η_p_*^2^ = 0.038.

As a supplementary analysis integrating response speed and accuracy, BIS showed a significant main effect of stimulus bias, *F*(1, 23) = 9.20, *p* = 0.006, *η_p_*^2^ = 0.286, with higher scores for P-biased (*M* = 0.236, *SD* = 1.520) than M-biased stimuli (*M* = −0.236, *SD* = 1.353), mean difference = 0.472, 95% CI [0.150, 0.794]. The main effect of stimulus category was also significant, *F*(1, 23) = 9.04, *p* = 0.006, *η_p_*^2^ = 0.282, with higher BIS for single-face than ensemble displays. The interaction was not significant, *F*(1, 23) = 1.68, *p* = 0.208, *η_p_*^2^ = 0.068. Bonferroni-corrected condition-wise comparisons further showed higher BIS for P-biased than M-biased stimuli in both the ensemble condition, *t*(23) = 2.84, adjusted *p* = 0.019, *d_z_* = 0.579, and the single-face condition, *t*(23) = 2.83, adjusted *p* = 0.019, *d_z_* = 0.577. Thus, the faster responses under P-biased stimulation were not offset by lower accuracy.

### 3.2. Multivariate Pattern Analysis Results

#### 3.2.1. Time-Resolved Decoding Results

**Figure 3:**
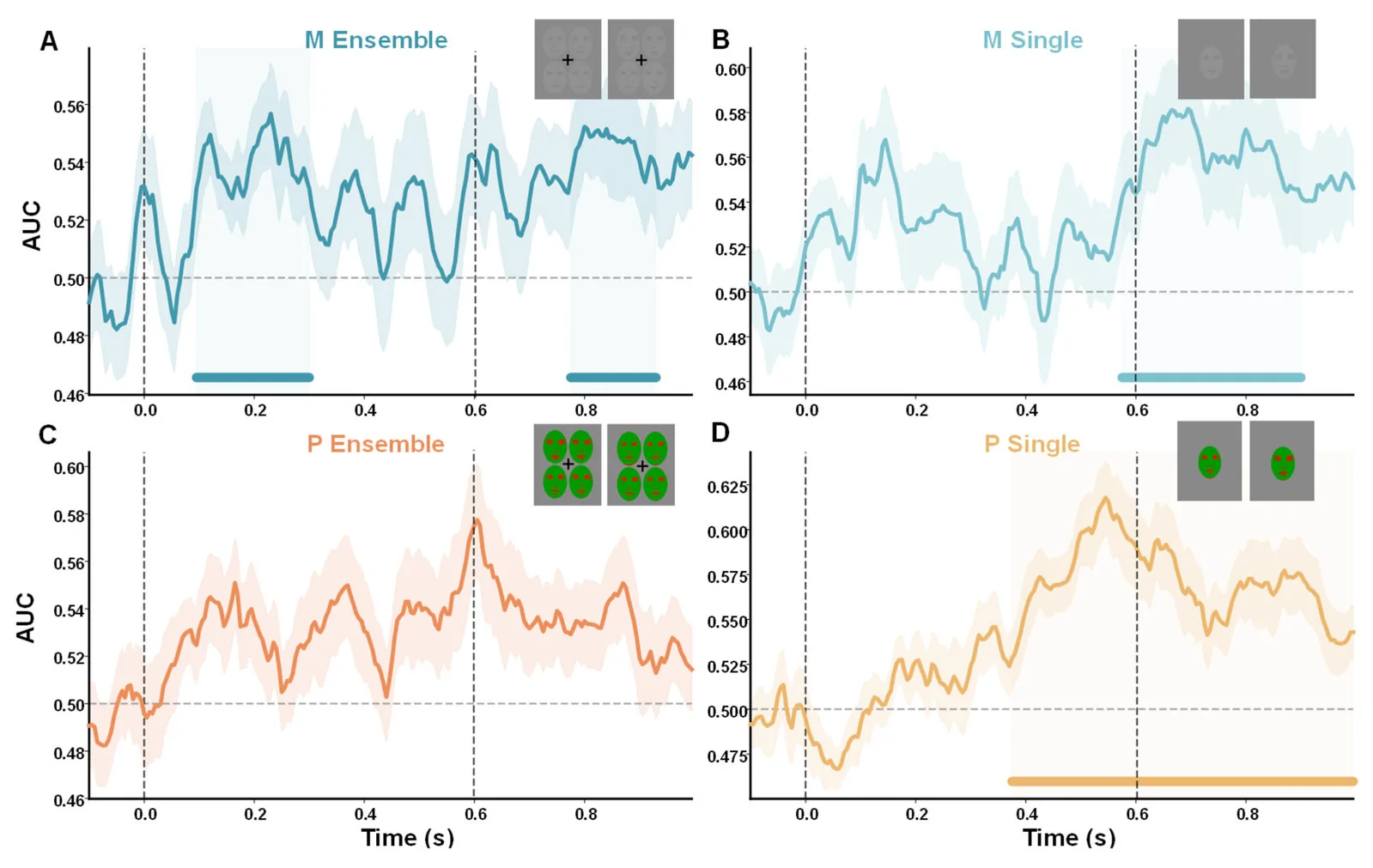
Time-resolved decoding of face-gender information in the four experimental conditions. (**A**) M-biased ensemble condition. (**B**) M-biased single-face condition. (**C**) P-biased ensemble condition. (**D**) P-biased single-face condition. Male-versus-female classification was performed separately within each condition. Curves show group-mean area under the receiver operating characteristic curve (ROC-AUC; *N* = 24), and shaded ribbons surrounding the curves indicate the SEM. Horizontal dashed lines indicate chance-level performance (AUC = 0.5), and vertical dashed lines indicate stimulus onset at 0 s and offset at 0.6 s. The stimuli remained on the screen from 0 to 0.6 s. Lightly tinted vertical bands and the corresponding colored horizontal bars mark significant temporal clusters identified by one-tailed cluster-based permutation tests with 5000 permutations, *p*_cluster_ < 0.05. Insets show representative masculine and feminine stimuli from the two categories entered into the classifier.

Time-resolved multivariate pattern analysis (time-resolved MVPA) was used to examine whether target information could be decoded from EEG signals under each condition (Figure 3). Classification performance was quantified using ROC-AUC, with a chance level of 0.5. Above-chance decoding was assessed using cluster-based permutation tests, with correction for multiple comparisons across time within each condition. AUC means, standard errors, and confidence intervals within the identified clusters are reported descriptively. In the M-biased ensemble condition, two significant temporal clusters were identified, spanning 95–300 ms (*p*_cluster_ = 0.008) and 775–930 ms (*p*_cluster_ = 0.019) after stimulus onset. The first cluster had a mean AUC of 0.540 (SEM = 0.009), with a mean difference from chance of 0.040, 95% CI [0.022, 0.058]. The second cluster had a mean AUC of 0.545 (SEM = 0.016), with a mean difference from chance of 0.045, 95% CI [0.012, 0.079]. In the M-biased single condition, the cluster-based permutation test identified one significant temporal cluster, spanning 575–900 ms (*p*_cluster_ = 0.002). This cluster had a mean AUC of 0.562 (SEM = 0.019), with a mean difference from chance of 0.062, 95% CI [0.022, 0.102].

Face-gender decoding was then analyzed in the P-biased conditions. In the P-biased ensemble condition, no temporal cluster survived cluster-level correction across time (all *p*_cluster_ ≥ 0.072). In the P-biased single condition, the cluster-based permutation test identified one significant temporal cluster, spanning 375–995 ms (*p*_cluster_ < 0.001). This cluster had a mean AUC of 0.571 (SEM = 0.013), with a mean difference from chance of 0.071, 95% CI [0.045, 0.097].

Taken together, cluster-corrected above-chance decoding was observed in the M-biased ensemble condition and in both single-face conditions. The M-biased ensemble condition showed an early significant cluster at 95–300 ms and an additional late cluster at 775–930 ms, whereas the significant clusters in the single-face conditions covered later intervals: 575–900 ms in the M-biased condition and 375–995 ms in the P-biased condition. Thus, target information could be reliably decoded from EEG signals in these three conditions, with the earliest significant temporal cluster descriptively observed in the M-biased ensemble condition.

**Figure 4:**
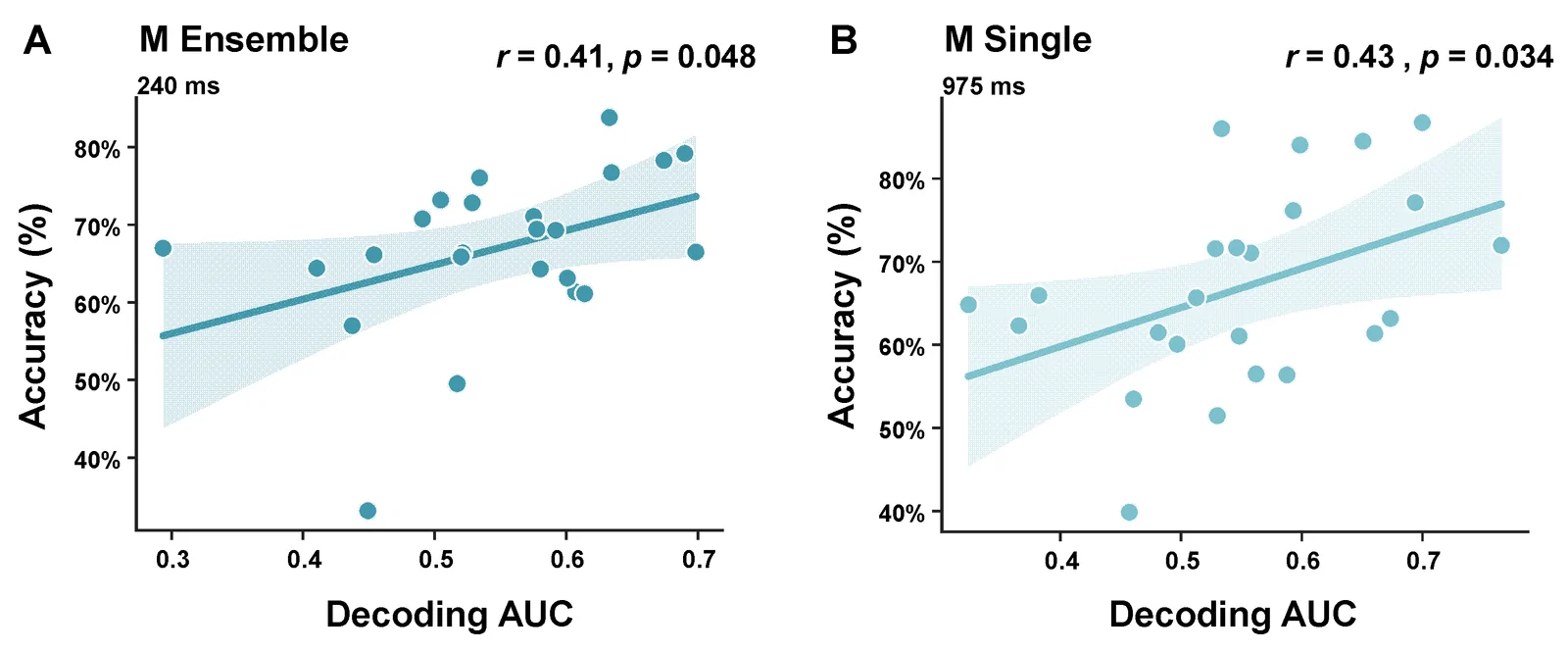
Participant-level associations between time-resolved neural decoding and behavioral accuracy in the M-biased conditions. (**A**) Association between decoding AUC at 240 ms and behavioral accuracy in the M-biased ensemble condition. (**B**) Association between decoding AUC at 975 ms and behavioral accuracy in the M-biased single-face condition. Each point represents one participant (*N* = 24). The x-axis shows participant-level face-gender decoding AUC at the indicated latency, and the y-axis shows behavioral accuracy in the corresponding condition. Solid lines show least-squares regression fits, and shaded bands indicate 95% confidence intervals around the fitted lines. Pearson correlation coefficients and their corresponding *p* values are displayed in the upper part of each panel.

Exploratory participant-level analyses showed positive associations between decoding AUC and behavioral accuracy at 240 ms in the M-biased ensemble condition, *r* = 0.41, and at 975 ms in the M-biased single-face condition, *r* = 0.43 (Figure 4). Both associations reached nominal pointwise significance with two-sided *p* values below 0.05 and are presented as exploratory results.

#### 3.2.2. Cross-Condition Generalization Decoding

**Figure 5:**
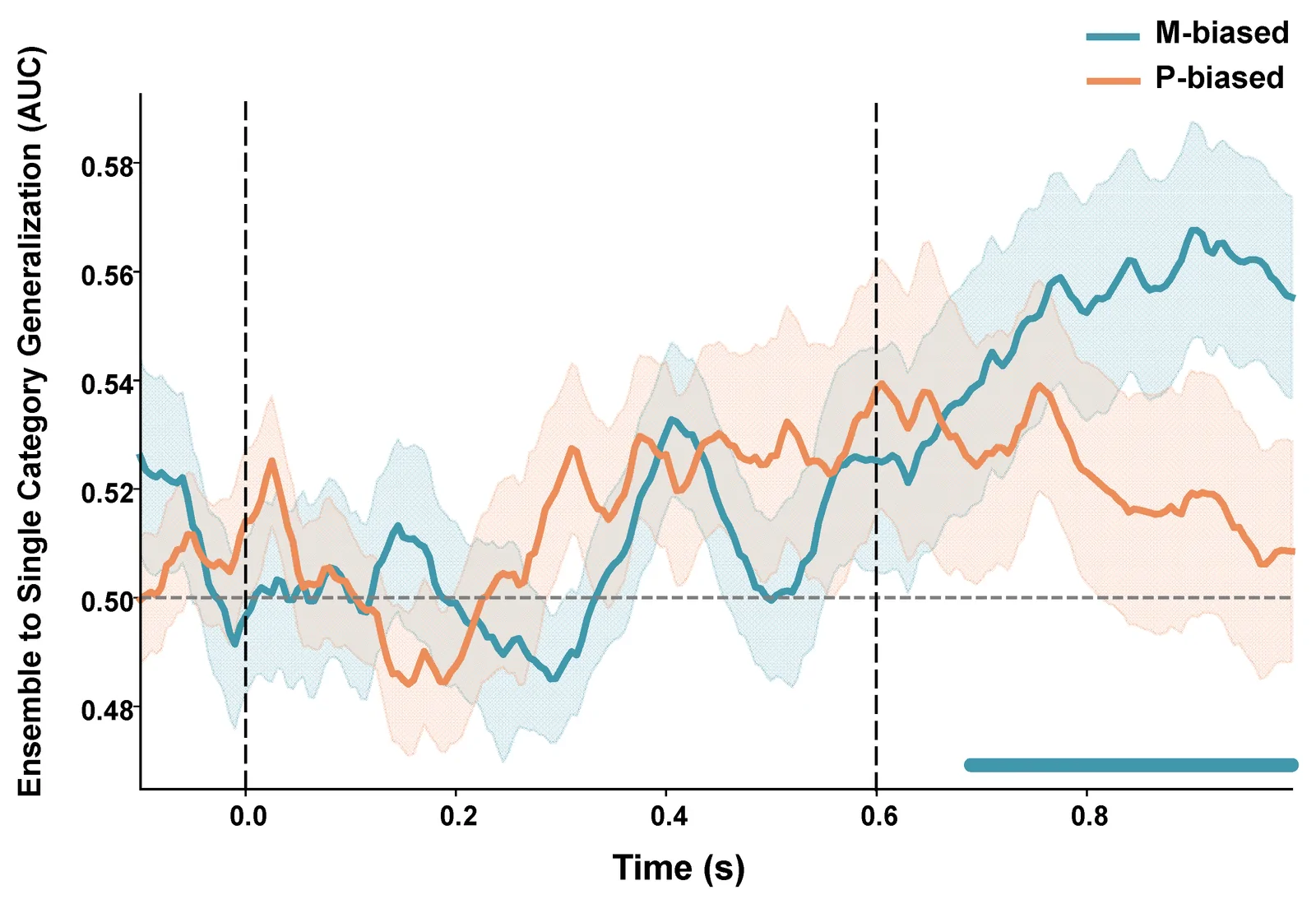
Time-resolved ensemble-to-single-face cross-condition generalization of face-gender information. Classifiers trained to distinguish masculine from feminine ensemble pseudotrials were tested on single-face pseudotrials at the corresponding analysis window, separately under M-biased and P-biased stimulation. Teal and orange curves show group-mean area under the receiver operating characteristic curve (AUC; *N* = 24), and shading indicates the standard error of the mean (SEM). The horizontal dashed line marks chance performance (AUC = 0.5); vertical dashed lines mark stimulus onset at 0 s and offset at 0.6 s. The teal horizontal bar indicates the significant M-biased generalization cluster identified by a one-tailed cluster-based permutation test with 5000 permutations, with correction across time and additional Bonferroni correction across the two stimulus-bias analyses (corrected *p*_cluster_ < 0.05).

Cross-condition generalization decoding was used to determine whether gender-discriminating information learned from ensemble trials could be transferred to single-face trials (Figure 5). Under M-biased stimulation, ensemble-to-single generalization yielded a significant late cluster spanning 690–995 ms, *p*_cluster_ = 0.0104 after correction across time and across the M- and P-biased analyses. The descriptive mean AUC within this cluster was 0.556 (SEM = 0.017), and the cluster extended to the end of the analyzed interval. Descriptively, the significant ensemble-to-single-face generalization cluster at 690–995 ms followed the early M-biased ensemble decoding cluster at 95–300 ms and overlapped with later decoding in both M-biased display conditions. This result identifies a late period during which gender-discriminating task information generalized from ensemble to single-face displays. Under P-biased stimulation, the same analysis did not identify a cluster meeting the corrected significance threshold.

#### 3.2.3. Temporal Generalization Decoding Results

Temporal generalization decoding was further used to analyze the temporal generalization patterns of ensemble and single faces. This analysis tested whether discriminative patterns learned by the classifier at different training time points could generalize to other testing time points and assessed the temporal stability of neural representations. Specifically, a classifier was trained at each training time window and evaluated at all testing time windows, yielding a two-dimensional training-time × testing-time decoding matrix. Statistical inference used a TFCE-enhanced permutation test with family-wise error (FWE) correction across the full two-dimensional matrix (5000 permutations). One-tailed group-level tests evaluated whether the mean logit-transformed AUC exceeded zero, corresponding to chance performance on the transformed scale. Before statistical analysis, AUC values were clipped to avoid infinite values in the logit transformation, so that chance level (AUC = 0.5) corresponded to 0 in the transformed space.

**Figure 6:**
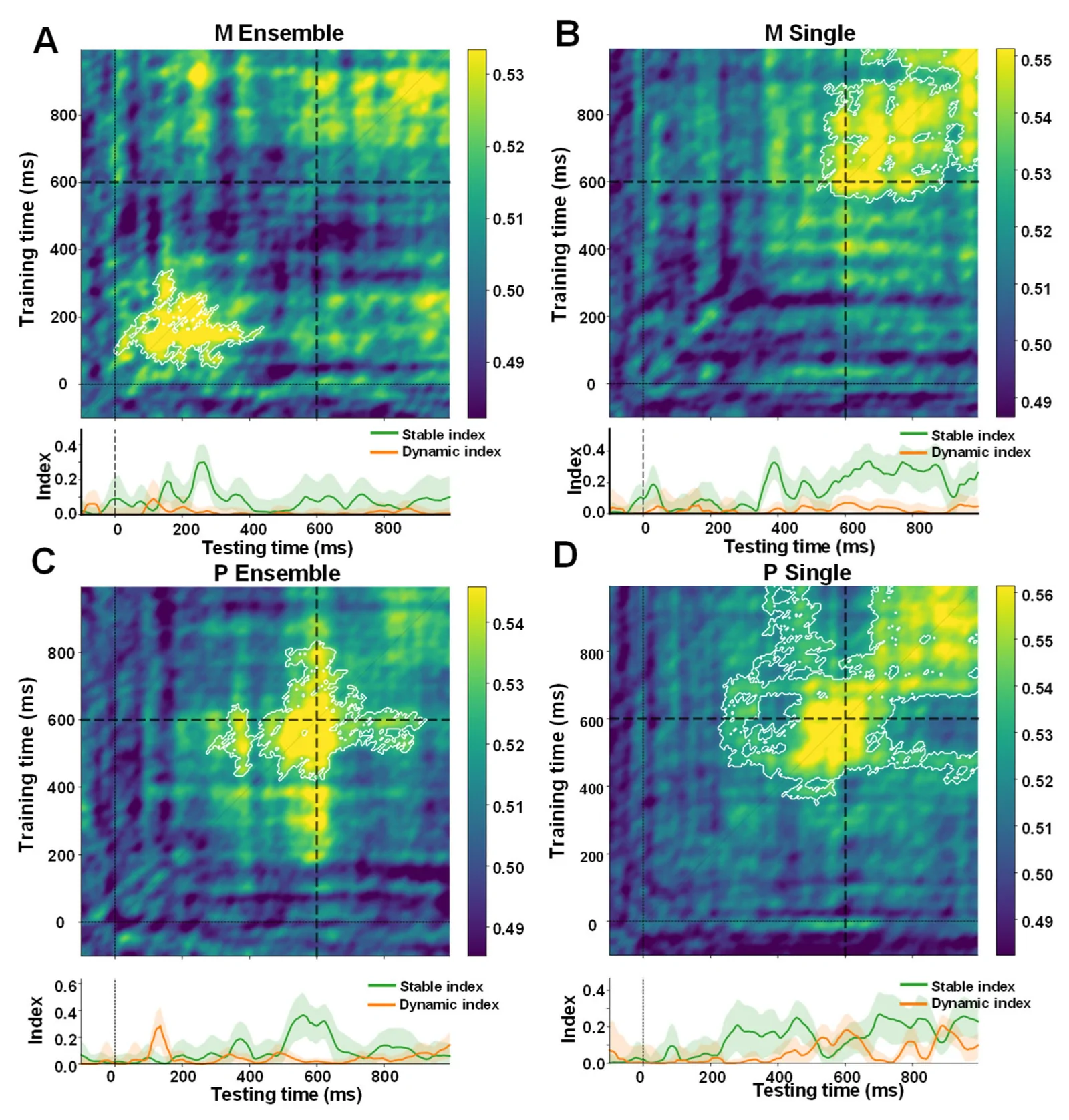
Temporal-generalization decoding of face-gender information and operationally defined stable and dynamic coding indices. (**A**) M-biased ensemble condition. (**B**) M-biased single-face condition. (**C**) P-biased ensemble condition. (**D**) P-biased single-face condition. Temporal-generalization matrices show group-mean ROC-AUC (*N* = 24), with training time on the y-axis and testing time on the x-axis. Color bars indicate ROC-AUC, with scales varying across panels. In each matrix, black reference lines mark stimulus onset (0 ms) and offset (600 ms) on both axes, and the diagonal indicates identical training and testing times. White contours indicate regions significantly above chance (*p*_FWE_ < 0.05, TFCE-based permutation corrected; 5000 permutations). The lower panels show the stable (green) and dynamic (orange) coding indices. Dynamic coding denotes off-diagonal decoding significantly lower than both corresponding diagonal values, whereas stable coding denotes above-chance off-diagonal decoding that was not significantly lower than either diagonal value. Shaded areas indicate ±1 bootstrap standard error estimated by resampling participants with replacement.

The temporal generalization results are shown in Figure 6. Significant regions for M-biased and P-biased stimuli showed different descriptive distributions for ensemble and single stimuli. In the M-biased ensemble condition, one reliable early significant region was detected in the two-dimensional decoding matrix (*p*_FWE_ < 0.05; Figure 6A). This region was mainly distributed across combinations of early training and early testing times, with training times of approximately 45–340 ms and testing times of approximately 10–440 ms. This pattern indicates that early ensemble face-gender information from M-biased ensemble stimuli could be read out and generalized across adjacent early time windows. The significant region for M-biased single stimuli was broader and was mainly distributed at later stages. In this condition, the significant region was distributed across training times of 540–995 ms and testing times of 520–995 ms (Figure 6B). Descriptively, significant generalization in the M-biased single condition occurred mainly during late processing and covered a broad range of training and testing times, consistent with shared gender-discriminative patterns across these late time windows.

In the P-biased conditions, temporal generalization patterns also showed different descriptive distributions for ensemble and single faces. After permutation testing, the P-biased ensemble condition showed one significant region at middle-to-late processing stages, with training times of 420–830 ms and testing times of 280–925 ms (Figure 6C). In the P-biased single condition, the significant region was concentrated mainly at middle-to-late processing stages, with training times of 345–995 ms and testing times of 225–995 ms (Figure 6D). Descriptively, gender-discriminative information for P-biased single faces was expressed mainly during middle-to-late stages and generalized across a broad range of time windows. The extent of the significant region was also broader than that in the P-biased ensemble condition.

The dynamic and stable indices provided a summary of the temporal organization of the generalization matrices. In the M-biased ensemble condition, variations in both indices were concentrated mainly during early processing and were relatively brief, suggesting limited early cross-temporal preservation accompanied by continuing temporal updating. In the P-biased ensemble condition, the stable index increased mainly during the middle-to-late period, broadly corresponding to the sustained generalization region in the temporal-generalization matrix. Under both single-face conditions, stable-index changes were concentrated primarily during middle-to-late processing, with intermittent dynamic-index changes indicating that temporal updating and cross-time preservation may coexist.

## 4. Discussion

By manipulating chromatic and luminance contrast to construct M-biased and P-biased facial stimuli, the present study combined an ensemble face gender-judgment task, high-temporal-resolution EEG recordings, time-resolved MVPA, and temporal generalization to investigate how ensemble average gender representations depend on pathway-biased visual inputs over time. Results showed that M-biased ensemble gender information was reliably decoded during early and late processing stages (95–300 ms and 775–930 ms), with significant temporal generalization across early time windows. P-biased ensemble faces showed no significant time-resolved decoding cluster, although temporal generalization emerged during middle-to-late processing.

M-biased visual information may contribute to the early availability of ensemble gender information. In the M-biased ensemble condition, gender information from face ensembles was reliably decoded during an early interval of 95–300 ms after stimulus onset, with significant temporal generalization between adjacent early time windows, suggesting that ensemble gender information exhibited a degree of temporal continuity at an early stage of perception. By contrast, significant decoding in the M-biased single-face condition was observed primarily during the later interval of 575–900 ms. Because decoding significance was assessed separately within the ensemble and single-face conditions, this difference in cluster timing should be interpreted descriptively. This temporal pattern is broadly consistent with previous electrophysiological evidence that discriminative neural activity related to ensemble mean information can emerge in the visual system as early as approximately 100 ms after stimulus onset [7,8,38]. It also accords with classic findings in ensemble perception that summary statistical information can be extracted rapidly, whereas fine-grained processing of individual members occurs relatively later [3,4]. One possible explanation comes from the model of top-down facilitation in visual recognition. According to this model, coarse information carried by low spatial frequencies can be rapidly transmitted to higher-order regions, generating preliminary predictions about the current visual input and constraining subsequent processing of finer details [12,13,14]. Given the high temporal sensitivity associated with M-biased input and its advantage in conveying coarse global structural information, such input may be particularly well suited to supporting the rapid integration of gender cues distributed across multiple faces. Previous studies have also found that face ensemble processing can engage dorsal visual regions associated with global information processing at relatively early stages, and that low-spatial-frequency information may facilitate the rapid processing of face ensembles [9,18]. At the physiological level, M-pathway neurons are highly sensitive to low luminance contrast, low spatial frequencies, and transient signals, and their axons conduct signals rapidly [14]. These properties make the M pathway well suited to rapidly conveying global structural cues and supporting coarse representations of ensemble averages during the initial stages of visual processing [15]. Thus, the early decoding observed in the present study is consistent with the formation of an initial estimate of ensemble gender from M-biased information and provides temporal evidence that M-biased visual input is available during early social ensemble processing.

The late decoding results further characterize the representation of M-biased ensemble information at later stages of processing. Under M-biased conditions, gender information from face ensembles and single faces was significantly decoded at 775–930 ms and 575–900 ms after stimulus onset, respectively, and a classifier trained on face ensembles also generalized to single faces at 690–995 ms. The three effects overlapped temporally at 775–900 ms, suggesting that ensemble and single-face judgments may draw on partially shared gender-discriminative task information during late processing. Because this common interval occurred after stimulus offset, its functional interpretation should remain cautious, as the shared information may include late-stage evaluation, evidence integration, decision formation, response preparation, and other task-related processes. Previous studies have shown that ensemble encoding can retain information about individual members and that finer-grained member representations may contribute to ensemble judgments [6,17]. Later recruitment of member-level cues therefore provides one possible account of the observed temporal overlap. The cross-condition generalization supports shared gender-discriminative task information across ensemble and single-face displays, while leaving the representational format of that shared information open. Together, the late temporal overlap and cross-condition generalization complement the early M-biased ensemble decoding effect by showing that gender-discriminative information remains available across later stages of processing.

Temporal generalization further characterized the temporal organization of these representations. For M-biased ensembles, significant generalization was concentrated during early processing, while the descriptive stable and dynamic indices suggested early cross-temporal preservation together with continuing temporal updating. P-biased ensembles showed significant generalization mainly during middle-to-late processing, accompanied by an increase in the stable index, while both single-face conditions also showed generalization primarily during middle-to-late processing. The P-biased ensemble pattern therefore provides complementary evidence from temporal generalization for middle-to-late cross-temporal readability. These patterns show that M-biased ensemble information was available during early processing and remained decodable at later stages. P-biased ensemble information showed sustained cross-temporal readability during middle-to-late processing. Related neuroscientific evidence also suggests that ensemble representations may continue to develop after their initial emergence. Using an inverted encoding model (IEM), Yashiro et al. [11] found that mean-orientation representations in heterogeneous ensembles gradually strengthened approximately 400–700 ms after stimulus onset and were significantly associated with subjective mean-orientation reports at 600–700 ms. Similarly, Gong et al. [10] reported that IEM-based neural activity related to mean orientation emerged from approximately 370 ms. Both studies used line orientation stimuli. Together with the early and late decoding effects observed for M-biased ensemble faces, these findings are consistent with the possibility that ensemble-related gender information remains available and changes in temporal organization across multiple processing stages.

Behavioral reaction times and neural decoding trajectories provide complementary perspectives on face-gender processing, highlighting the value of time-resolved electrophysiology in characterizing the temporal availability of gender-related information. Behaviorally, responses were slower for ensemble than single faces and faster for P-biased than M-biased stimuli, with no significant accuracy differences. The BIS results further showed significantly higher scores for P-biased than M-biased stimuli in both ensemble and single-face conditions, indicating a more favorable combination of response speed and accuracy under P-biased stimulation. Although gender-related information in M-biased ensembles was decodable early, this pattern is consistent with the possibility that low-contrast M-biased inputs support the rapid extraction of coarse ensemble gender information, while additional processing may still be required for explicit decisions. Exploratory participant-level correlations at 240 ms for M-biased ensembles and 975 ms for M-biased single faces provide preliminary support from an individual-differences perspective for a relationship between neural discriminability and behavioral performance. In contrast, the richer chromatic and local facial information in P-biased stimuli may provide clearer diagnostic cues for explicit judgments, potentially contributing to faster responses. Together, these findings suggest that early M-biased ensemble decoding and the behavioral advantage for P-biased stimuli may reflect different stages of the processing cascade.

Methodologically, the present study used several controlled stimulus manipulations to compare the temporal dynamics of M- and P-biased visual inputs. Previous studies often relied on spatial-frequency filtering, although the substantial overlap in M- and P-neuron spatial-frequency tuning limits the specificity of this approach [19,20]. Here, low-luminance-contrast grayscale faces and isoluminant red–green faces were used to bias processing toward M- and P-related inputs, respectively, with red–green isoluminance empirically calibrated using heterochromatic flicker photometry [21,22,23]. Face luminance and contrast were further standardized, and multiple independent male–female morph continua, controlled ensemble composition, and counterbalanced spatial positions reduced physical and positional variability across conditions. In addition, using neutral facial gender rather than emotional expressions reduced potential contributions from affective arousal and threat-related processing, which can preferentially engage rapid magnocellular-biased or subcortical mechanisms [18,26,27,28,29]. Together, these manipulations allowed the temporal patterns to be interpreted more specifically in relation to ensemble gender processing under pathway-biased visual stimulation, while remaining limited to relative M- and P-biased inputs.

Despite clarifying the temporal dynamics of ensemble average representations, several limitations warrant consideration. First, our psychophysical manipulations induced relative biases rather than absolute isolation of the M and P pathways. Second, scalp EEG provides limited spatial specificity; future studies combining high-spatial-resolution fMRI or MEG source localization could further identify the neural substrates associated with M-biased and P-biased ensemble processing across dorsal, ventral, and prefrontal networks. Finally, the present findings are based on a limited set of Caucasian face identities and a participant sample with an unequal sex distribution, which may constrain their generalizability across face identities and observer populations. Future work should test whether the observed temporal patterns extend to more diverse face sets, participant samples, and other ensemble dimensions such as orientation, size, and color.

## 5. Conclusions

The present study characterizes the temporal organization of ensemble face-gender information under M-biased and P-biased visual stimulation. M-biased ensemble gender information was reliably decodable during early processing and generalized across early time points. P-biased ensemble information showed significant temporal generalization during middle-to-late processing. Later decoding of single faces and late ensemble-to-single generalization under M-biased stimulation further showed that gender-discriminative information remained available during later stages of the task. Together, these findings provide a time-resolved account of how social ensemble gender information is expressed under pathway-biased visual inputs and support a multistage view of ensemble processing.

## Data Availability

The raw data supporting the conclusions of this article will be made available by the authors upon reasonable request.
